# Does discontinuation of the use of hydroxyethyl starches in the critically ill cardiac surgery patient have an impact on caloric intake?

**DOI:** 10.1186/cc14477

**Published:** 2015-03-16

**Authors:** E De Waele, K De Bondt, S Mattens, J Czapla, J Nijs, M La Meir, D Nguyen, PM Honoré, H Spapen

**Affiliations:** 1 Universitair Ziekenhuis Brussel, Brussels, Belgium

## Introduction

After research revealed unwanted effects of the use of starches in critically ill patients, its use in the immediate postoperative period of cardiac surgery patients came to an abrupt ending. However, they constitute an important source of non-intended calories, providing 4 calories per gram. We investigated whether this phenomenon (involuntary) attributed to an increase in caloric debt for this critically ill patient population.

## Methods

We retrospectively searched a database of 417 elective cardiac surgery patients, representing 5,004 observation-days. Caloric intake was evaluated in the group of patients before and after the cessation of starch use.

## Results

Patient characteristics and caloric needs were comparable: 2,054 ± 395 kcal/day and 2,056 ± 347 kcal/day. The 140 patients who in the immediate postoperative period had volume resuscitation without the use of starches had a mean non-intended fluid caloric intake of 69 (± 36.3) kcal/day. The group of 277 patients who received starches in the postoperative period had a mean non-intended fluid caloric intake of 105 (± 100.2) kcal/day.

## Conclusion

Withdrawal of the use of starches resulted in a 34% decrease of non-intended caloric intake by fluids, contributing to caloric debt. Whether outcome is influenced and/or whether these findings are clinically relevant needs further research.

